# A Minimal Fragment of MUC1 Mediates Growth of Cancer Cells

**DOI:** 10.1371/journal.pone.0002054

**Published:** 2008-04-30

**Authors:** Sanjeev Mahanta, Shawn P. Fessler, Jaehong Park, Cynthia Bamdad

**Affiliations:** Minerva Biotechnologies, Waltham, Massachusetts, United States of America; Dresden University of Technology, Germany

## Abstract

The MUC1 protein is aberrantly expressed on many solid tumor cancers. In contrast to its apical clustering on healthy epithelial cells, it is uniformly distributed over cancer cells. However, a mechanistic link between aberrant expression and cancer has remained elusive. Herein, we report that a membrane-bound MUC1 cleavage product, that we call MUC1*, is the predominant form of the protein on cultured cancer cells and on cancerous tissues. Further, we demonstrate that transfection of a minimal fragment of MUC1, MUC1*_1110_, containing a mere forty-five (45) amino acids of the extracellular domain, is sufficient to confer the oncogenic activities that were previously attributed to the full-length protein. By comparison of molecular weight and function, it appears that MUC1* and MUC1*_1110_ are approximately equivalent. Evidence is presented that strongly supports a mechanism whereby dimerization of the extracellular domain of MUC1* activates the MAP kinase signaling cascade and stimulates cell growth. These findings suggest methods to manipulate this growth mechanism for therapeutic interventions in cancer treatments.

## Introduction

MUC1 is a Type I membrane glycoprotein of the mucin family having an extensive extracellular domain consisting of hundreds of tandem repeat units, a single transmembrane domain and a C-terminal cytoplasmic tail [1]–[5]. MUC1 is normally expressed at the apical border of healthy epithelia that line the respiratory, reproductive and gastrointestinal tracts. In sharp contrast to the healthy pattern of expression that restricts MUC1 to luminal surfaces, cancerous tissues display an aberrant expression pattern wherein MUC1 is uniformly distributed over the entire tissue surface [6], [7]. It is currently estimated that 75% of all human solid tumor cancers aberrantly express the MUC1 protein [8].

The function of MUC1 in the healthy state remains unclear, while evidence is rapidly accumulating for its role as an oncoprotein. The introduction of MUC1 into previously MUC1-negative cells results in an increased growth rate [9], enables anchorage-independent cell growth [10] and renders cells resistant to apoptosis induced by treatment with standard chemotherapy agents [8]. Interactions between MUC1 and members of the ErbB family have been reported [11], [12]. MUC1 is involved in several intracellular signaling pathways. These studies have focused on the MUC1 cytoplasmic tail (MUC1-CT), which is perhaps the best characterized portion of the protein [13]. The 72 amino acid tail bears residues that can be phosphorylated by Zap70, PCKδ, GSK3β, ErbB1, c-Src, Lyn, and Lck. In addition, signal transduction elements AP-2, p53, ER-α, β-catenin and Grb2 have been shown to bind to the MUC1-CT, presumably as a function of its phosphorylation state. Indeed, studies implicate the phosphorylation of the MUC1-CT in the activation of the MAP kinase signaling pathway [12]. In one such study [14], antibody stimulation of the extracellular domain of a chimeric protein comprised of the extracellular and transmembrane portions of CD8, but the cytoplasmic tail of MUC1, resulted in the phosphorylation of ERK 1/2. ERK activation was shown to be dependent on the phosphorylation of the MUC1-CT and could be abolished by a dominant negative Ras mutant or by a MEK inhibitor, thus arguing that the MUC1-CT mediates the Grb2-SOS-Ras-MEK-ERK signaling cascade which leads to cell division.

Studies of the MUC1 extracellular domain (ECD) are complicated by its shear size (150–300 kDa), the fact that it is highly glycosylated, and that it is made up of a variable number of tandem repeats. In addition, the protein can be cleaved, then shed from the cell surface. Shed MUC1, comprised largely of tandem repeats, can be detected in the serum of Stage II breast cancer patients [15]. Similarly, western blots of the lysates of MUC1-positive cultured cancer cells show two MUC1 species: a high molecular weight species (150–300 kDa) that reacts with antibodies that bind to the tandem repeats and a low molecular weight species (20–35 kDa) that reacts with antibodies that bind to the cytoplasmic tail. Cell supernatants contain only the high molecular weight MUC1 species. Some studies provide evidence that MUC1 self-cleaves via an SEA domain within the protein [16], [17]. Others have presented evidence that TACE (ADAM 17) and MMP14 (MT1-MMP) cleave MUC1 [18], [19]. Early reports postulated that after MUC1 cleavage, the released, high molecular weight portion re-associates with the membrane bound low molecular weight fragment, thus becoming the ligand for the membrane-bound receptor [20].

The present report focuses on the expression and function of the low molecular weight MUC1 cleavage product that remains membrane-bound after the bulk of the extracelluar domain has been cleaved and released from the cell surface.

## Results

### Characterization of a novel MUC1 antibody

**Figure 1:**
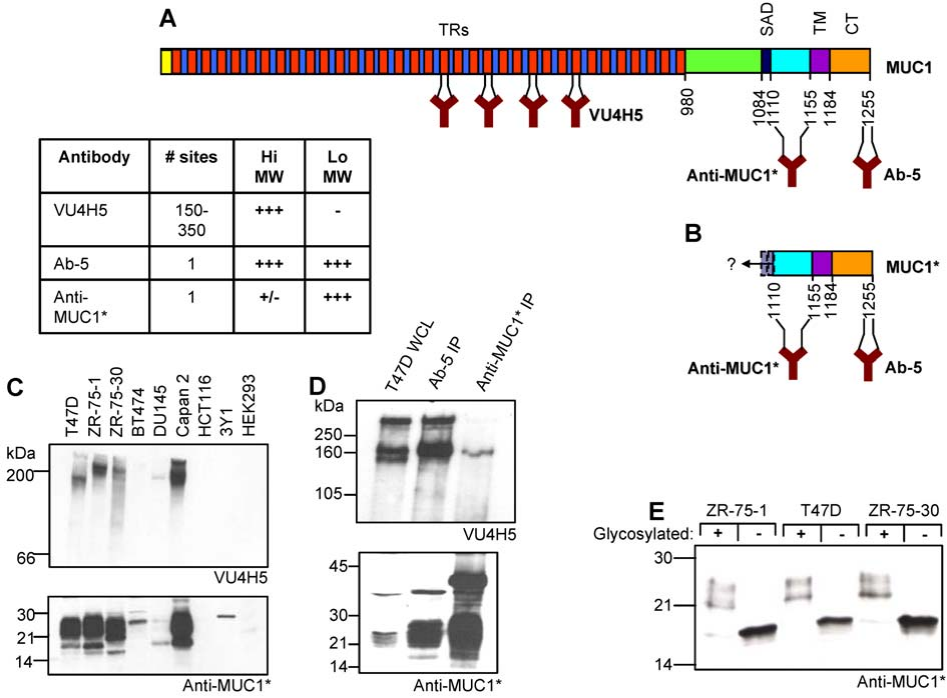
Schematic and antibody recognition of full-length MUC1 versus the membrane-bound cleavage product MUC1*. A. Full-length MUC1 protein is comprised of a cytoplasmic tail (CT), a transmembrane domain (TM), a self-aggregation domain (SAD), and hundreds of tandem repeats (TRs). B. Cleavage product, MUC1*, consists of the cytoplasmic tail, transmembrane domain, and at least 45 amino acids of the extracellular domain (ECD). Although the exact site(s) of cleavage remain somewhat uncertain, to our knowledge, no cleavage sites have been reported that leave less than a 45 amino acid ECD. Binding sites for antibodies VUH5, Ab-5 and Anti-MUC1* are marked. C. Western analysis of whole cell lysates of MUC1-positive cultured cancer cells, Lanes 1–6, are compared to MUC1-negative cells, Lanes 7–9. A 6% gel (upper) was blotted with VU4H5 and a 12% gel (lower) was blotted with Anti-MUC1*. D. T47D, MUC1-positive cultured breast cancer cells, were immunoprecipitated with either Ab-5 or Anti-MUC1*. Samples of the whole cell lysate (WCL), Lane 1, or the immunoprecipitates, Lanes 2 and 3, were analyzed by western blot. Gels were probed with either VU4H5 (upper) or Anti-MUC1* (lower). E. Western analysis of three MUC1-positive breast cancer cell lines pre- and post- deglycosylation and blotted with Anti-MUC1* shows that the actual molecular weight of cleaved MUC1 is approximately 16–18 kDa. F. The table summarizes the reactivities of antibodies to either full-length MUC1 (Hi MW) or cleaved MUC1 (Lo MW).

In order to investigate the expression patterns of MUC1 and its cleavage product as well as their respective functions, we needed to develop antibodies that could distinguish the low molecular weight cleavage product from the full-length protein on intact cells and tissue specimens. Antibodies that recognize the tandem repeat motifs (VU4H5, Santa Cruz) and the cytoplasmic tail (Ab-5, LabVision) are commercially available (Fig. 1A). However, antibodies that recognize the extracellular portion of the membrane-bound cleavage product are not. To complicate matters, the exact site(s) at which MUC1 is cleaved on cancer cells is unclear. Several cleavage sites for MUC1 have been reported or postulated [21], [22]. In fact, one of the enzymes that cleaves MUC1, MMP14, can cleave its substrates at multiple sites [23]. However, no cleavage site that has been reported or proposed would produce a membrane bound fragment with an extracellular domain shorter than forty-five (45) amino acids. Therefore, we raised an antibody against the forty-five (45) amino acid peptide (N-1110-1154-C) that is immediately N-terminal to the transmembrane domain (Fig. 1B). We reasoned that this antibody would recognize the membrane-bound products of all reported or proposed cleavage sites. Due to the uncertainty of the precise position of MUC1 cleavage, herein, we generically refer to all membrane-anchored MUC1 cleavage products as MUC1*. Similarly, we refer to antibodies raised against the forty-five amino acid peptide (N-1110-1154-C) as Anti-MUC1*. Both polyclonal and monoclonal antibodies were generated and they essentially performed equivalently.

Anti-MUC1* antibodies were characterized by western blot, FACS, and immunocytochemistry. Figure 1C shows a western blot in which cell lysates from a panel of MUC1-positive cultured cancer cells were probed with either VU4H5 (upper gel) or Anti-MUC1* (lower gel). As expected, VU4H5 stained the high molecular weight MUC1 species from all MUC1-positive cells tested: T47D, ZR-75-1, ZR-75-30, BT474, DU145, and Capan 2. Anti-MUC1* stained a low molecular weight 20–35 kDa protein band that appeared to be specific for MUC1. MUC1-negative cells, HCT116, 3Y1, and HEK293, did not react with either antibody. To confirm that the low molecular weight species that reacted with Anti-MUC1* was indeed MUC1, lysates from MUC1-positive tumor cells were immunoprecipitated with either Ab-5 (cytoplasmic tail antibody) or Anti-MUC1*, then analyzed by western blot (Fig. 1D). Anti-MUC1* binds to the same low molecular weight species that Ab-5 recognizes, thus confirming that Anti-MUC1* binds to the low molecular weight MUC1 cleavage product.

As previously reported, by western blot analysis, the high molecular weight MUC1 species only reacted with antibodies directed against the tandem repeats, and did not react with Anti-MUC1* or Ab-5 (data not shown). However, both antibodies are able to weakly immunoprecipitate the high molecular weight species (Fig. 1D, upper gel). In these experiments, immunoprecipitation may simply bring down a non-covalently associated hetero-dimer of the two cleavage fragments. These results might also be explained by the fact that VU4H5 binds to a tandem repeat unit that is repeated hundreds of times per receptor while Anti-MUC1* and Ab-5 bind to non-repeating sequences that occur only once per receptor. Alternatively, these results might simply be explained by the fact that the major species on cancer cells is the cleaved form, MUC1*. The table of Figure 1 summarizes the antibody binding data.

To further explore the actual size of the low molecular weight MUC1 species, cell lysates were deglycosylated prior to western blot analysis. Figure 1E shows that after deglycosylation, the previously broad 20–35 kDa protein band converges to a discrete band that runs with an apparent molecular weight of about 15–17 kDa. We note that the calculated molecular weight of a MUC1 cleavage product, trunacted after about forty-five amino acids of the extracellular domain is approximately 16 kDa.

### Expression Patterns of MUC1 and its Cleavage Product, MUC1*, on Cultured Cells and on Cancerous Tissues

**Figure 2:**
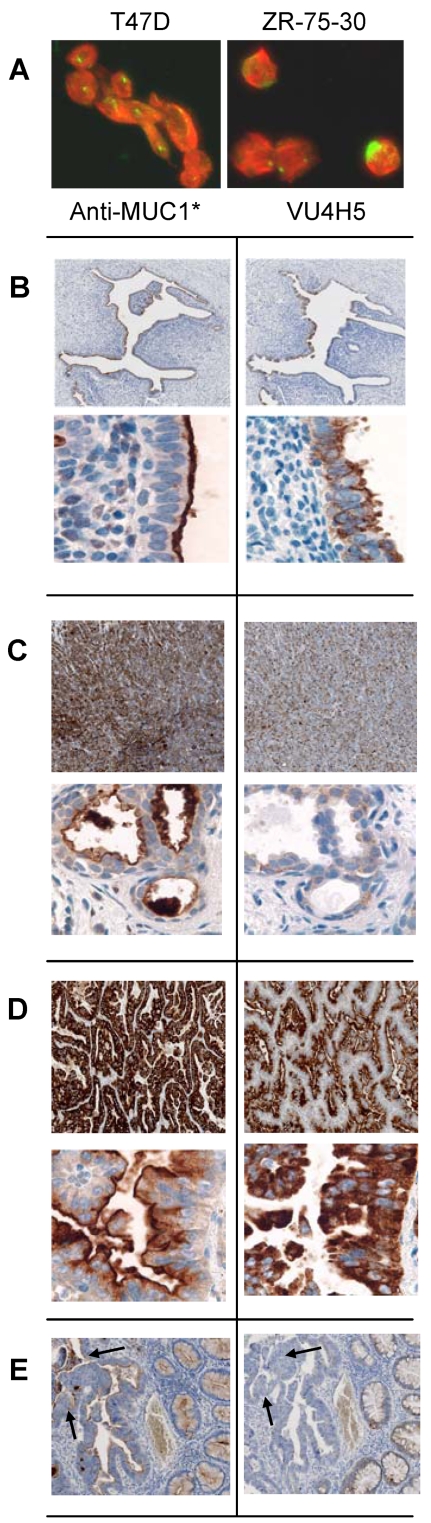
MUC1* is the major species on cultured cancer cells and on human tumor tissue. A. Fluorescent imaging of cultured breast cancer cells shows that the cleavage product MUC1* (red) is the predominant species and is uniformly distributed over the entire cell surface. Full-length MUC1 (green) is the minor species and is clustered. Cells were double stained with Anti-MUC1* (red) that binds to an epitope within the membrane proximal first 45 amino acids of the extracellular domain and VU4H5 (green) that binds to an epitope within the tandem repeat domain of the full-length protein. B–E. Pairs of contiguous sections of human tissue specimens from untreated patients were stained with either Anti-MUC1* (left) or VU4H5 (right). Images were photographed at 10× or 100× magnification. B. A cross section of a non-cancerous fallopian tube shows MUC1* surface staining at the luminal edge (lower left). Full-length MUC1 is cytoplasmic (lower right). C. Breast cancer tissue specimens. D. Lung cancer specimens. E. Colon cancer specimens. Arrows point to the most involved portions of the cancerous tissue where all cellular architecture has been lost and tissue is not stained by VU4H5.

Cultured cancer cells were analyzed by immuno-cytochemistry to determine the expression pattern of MUC1 and its cleavage product MUC1*. MUC1-positive breast cancer cells were double stained with VU4H5 that binds to tandem repeats units in the high molecular weight fragment and Anti-MUC1* that binds to the low molecular weight, membrane-bound cleavage product, MUC1*. Fluorescent imaging revealed that MUC1* is the predominant form of protein on cultured cancer cells and is uniformly distributed over the entire cell surface. In contrast, proteins that were stained by VU4H5 were either not present at all or were the minor species (Fig 2A). VU4H5 staining could indicate that the protein on the surface is full-length MUC1 or that it is a non-covalently bound hetero-dimer consisting of both cleavage products. Notably, the pattern of expression of full-length MUC1 is clustered at one or two points on the cell surface, which is reminiscent of the tendency of MUC1 on healthy epithelium to cluster at the apical border.

We next looked at the expression pattern of full-length MUC1 versus MUC1* healthy and cancerous human tissue specimens from untreated patients. Serial sections of a non-cancerous fallopian tube were stained with either Anti-MUC1* or VU4H5. Both MUC1 antibodies stained the luminal surface of the fallopian tube but not the surrounding tissue (Fig. 2B, upper panels). 100-fold magnification revealed that the cleaved form, MUC1*, is exclusively expressed on the *surface* of the epithelial cells lining the tube, while the full-length MUC1 protein appears to be limited to the cytoplasm (Fig. 2B, lower panels). Back-to-back sections of cancerous breast, lung, and colon tissues were similarly probed with the two antibodies (Figs. 2C–E, respectively). In sharp contrast to the expression pattern of MUC1 on healthy tissues, which was confined to the luminal edge, MUC1 is expressed over the entire surface of cancerous tissues. Staining with Anti-MUC1* shows that, like on cultured cancer cells, the major form of MUC1 on cancerous tissues is MUC1*. Magnified images show that MUC1* is expressed on the cell *surface* while full-length MUC1 appears to be cytoplasmic (Figs. C, D, lower panels). Notably, Anti-MUC1* produced heavier staining than VU4H5 despite the fact that the tandem repeat antibody can bind to hundreds of epitopes per receptor compared to the single epitope to which Anti-MUC1* binds. Some of the cancerous tissues that we tested did not stain positive with either MUC1 antibody. Of nine (9) breast cancer specimens tested, only one was a MUC1-negative cancer, which roughly corresponds to published reports that approximately 90% of all breast cancers are MUC1-positive cancers. We note that tissue specimens that were deemed to be MUC1-negative cancers showed normal apical staining with both MUC1 antibodies in regions outside of the margin of malignancy (data not shown) confirming that the assay was correctly performed but that these cancers were not characterized by aberrant expression of MUC1.

Importantly, we noticed that some tissue specimens stained positive for the presence of MUC1* but negative for full-length MUC1. For example, a colon cancer specimen was stained by Anti-MUC1* with the most intense staining occurring in the most diseased portions of the specimen. In contrast, VU4H5 lightly stained areas close to the margin of malignancy but produced no staining in the more diseased regions, characterized by the loss of cellular architecture (Fig. 2E). These results are consistent with the idea that the most cancerous specimens may appear to be MUC1-negative if probed with antibodies against the full-length protein alone. Taken together, these results demonstrate that the predominant MUC1 species on cancerous tissue, as on cultured cancer cells, is the cleaved species, MUC1*.

### MUC1* mediates cell growth

After determining that the major species on the surface of cancer cells and tissues is MUC1* and not the full-length protein, we decided to study the growth characteristics of the cleaved form compared to uncleaved. MUC1-negative cells, 3Y1 and HCT116, were transfected with either full-length MUC1 or a construct, MUC1*_1110_, whose extracellular domain was terminated after forty-five (45) amino acids (N-1110-1255-C) (Fig. 1B). Stable transfectants turned out to be inadequate for study because the full-length transfectants quickly evolved into a population dominated by the cleaved form, MUC1*, having little or no full-length protein available on the cell surface (data not shown). For this reason, single cell clones were generated and used in the experiments described in the present study. FACS, immunocytochemistry and Western blot analysis showed that our single cell clones of full-length MUC1 produced the full-length protein but also generated the cleavage product MUC1* (Fig. S1).

**Figure 3:**
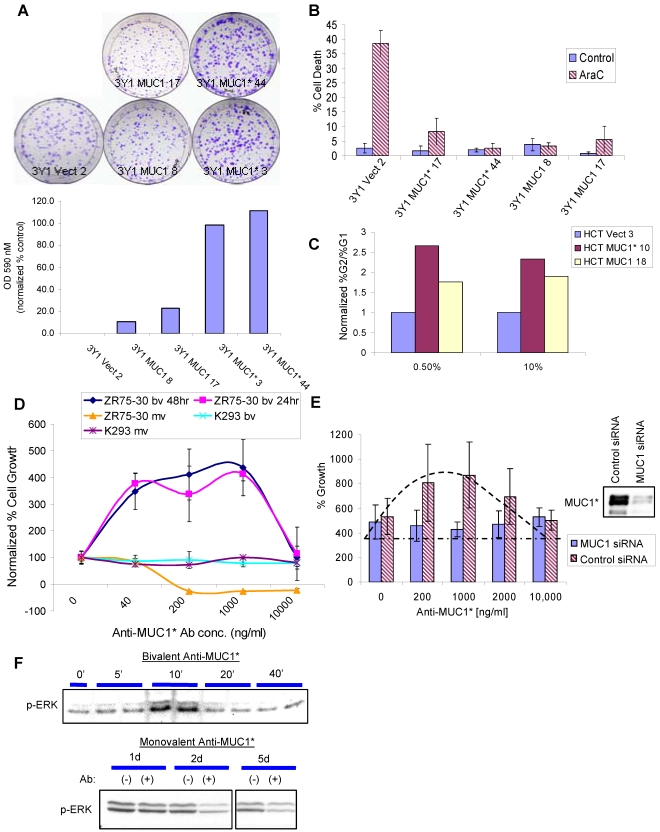
MUC1* mediates growth factor receptor-like activity. A. A clonogenic assay was performed on single cell clones of MUC1-negative 3Y1 cells that had been transfected with either an empty vector, full-length MUC1 or MUC1*_1110_. A photograph of Petri dishes containing cells plated at 1000 cells per 100 mm, grown for 9 days, then stained with crystal violet. Bar graph quantifies growth of each clone. Crystal violet absorbed by the cells on each plate was released and absorbance at 590 nm was measured on a spectrophotometer. Absorption measurements were normalized to the amount of crystal violet released from the empty vector clone. Nomenclature for our single cell clones is “parent cell name/construct/clone #”. B. Resistance to cell death induced by the chemotherapy drug AraC is tested in single cell clones of either empty vector, full-length MUC1 or MUC1* transfected into 3Y1 cells. C. Cell cycle analysis was performed on either empty vector, full-length MUC1 or MUC1* transfected into MUC1-negative cell line HCT116. The ratio of the percentage of cells in G2 to G1 phase is plotted as a function of serum concentration. The ratio of %G2∶%G1 for the empty vector transfectants has been normalized to 1. D. The growth of MUC1-positive breast cancer cells, ZR-75-30, is stimulated by the addition of bivalent (bv) Anti-MUC1* and inhibited by the addition of the monovalent (mv) Fab. The addition of bivalent antibody produces the bell-shaped growth curve that is characteristic of receptor dimerization. The growth of MUC1-negative HEK 293 cells was not impacted by either the bivalent or monovalent Anti-MUC1*. E. The stimulation of growth by the addition of bivalent Anti-MUC1* is tested using stably transfected siRNA to suppress MUC1 expression in breast cancer cell line T47D. Bivalent Anti-MUC1 stimulated growth in cells transfected with control siRNA, but did not significantly effect growth in the MUC1 suppressed cells. Western blot analysis (insert) shows that MUC1 siRNA suppressed MUC1 expression by about 90%. F. MUC1-positive breast cancer cells, T47Ds, are treated with either bivalent Anti-MUC1* or the monvalent Fab, then analyzed by Western for the presence of phosphorylated ERK1/2. Blots show the induction of ERK2 phosphorylation by bivalent Anti-MUC1* and inhibition of basal ERK1/2 phosphorylation after two days (2 d) treatment with the monovalent Fab.

A clonogenic assay was performed to assess the growth rate of MUC1*_1110_ versus full-length MUC1 that had been transfected into rat fibroblast 3Y1 cells. Cells plated at low density were allowed to grow for nine (9) days. Resultant colonies were visualized by staining with crystal violet. MUC1*_1110_ clones, 3Y1 MUC1* 3 and 3Y1 MUC1* 44, produced more, bigger and denser colonies than full-length clones, 3Y1 MUC1 8 and 3Y1 MUC1 17 (Fig. 3A). At the end of nine days, the MUC1*_1110_ clones produced five to ten-times more cells than the full-length clones. The difference in growth was quantified by measuring the amount of crystal violet that was released from the cells on each plate (Fig. 3A: bar graph). However, the increase in the number of cells could have been due to an increase in the survival rate or an increase in the growth rate. Cell survival factors can be identified by their ability to render cells resistant to chemotherapy-induced death. Single cell clones of MUC1 or MUC1*_1110_ that had been transfected into MUC1-negative cells (3Y1 or HCT116) were treated with AraC, cisplatin or etoposide, then assayed to measure the amount of cell death. Cells transfected with either full-length MUC1 or MUC1*_1110_ became resistant to apoptosis induced by these chemotherapy agents. A representative experiment is shown in Figure 3B. These results argue that MUC1 or MUC1* increase cell survival. To determine whether they also increase cell growth, a cell cycle analysis experiment was performed. FACS was used to measure the number of cells in G2/M phase (an indicator of cell division) compared to the number in G1 phase. The ratio of cells in G2:G1 was measured for HCT116 cells transfected with either full-length MUC1 or MUC1*_1110_ (Fig. 3C). The introduction of either MUC1*_1110_ or full-length MUC1 drove more cells into the G2/M phase than the control transfection of the empty vector. However, transfection MUC1*_1110_ increased the ratio of G2∶G1 more than the transfection of the full-length proetin. Recall that although cells are transfected with the full-length protein, a considerable amount is proteolyzed to the MUC1* form.

Thus, having established a link between MUC1* and growth, we speculated that it may function as a growth factor receptor. Without a known ligand for MUC1 or MUC1*, it would be difficult to test the hypothesis. However, Class I growth factor receptors trigger cell growth via the dimerization of the extracellular domain of the receptor. We did have a bivalent antibody against the MUC1*_1110-ecd (extracellular domain)_ portion, which could be used to simulate its ligand. Others had previously reported that stimulation of this class of growth factor receptors with dimerizing antibodies generated bell-shaped growth curves [24]–[26]. This is because cell growth increases with increasing antibody concentration until maximal growth is achieved when one antibody dimerizes every two receptors. However, as the concentration of antibody goes to excess, each antibody binds to one receptor, so no dimerization occurs, and cell growth falls off. We performed similar experiments by treating MUC1-positive cancer cells as well as MUC1 and MUC1*_1110_ transfected cells with Anti-MUC1*. As a negative control, we treated cells with the monovalent Fab of Anti-MUC1*, which would not be able to dimerize the receptors. For each MUC1-positive or MUC1*_1110_-positive cell line that we tested, the bivalent antibody stimulated cell growth and produced the expected bell-shaped growth curve. In stark contrast, the monovalent Fab not only did not stimulate cell growth, but it induced cell death. The results of one such experiment is shown in Figure 3D, where the growth of MUC1-positive breast cancer cells, ZR-75-30s, is stimulated by Anti-MUC1* and inhibited by the monovalent Fab. MUC1-negative HEK293 cells are unaffected by either antibody. Control antibodies, whether bivalent or monovalent, had no effect on the growth of MUC1-positive tumor cell growth (Fig. S2). To confirm that the antibody-stimulation of cell growth was specifically mediated by MUC1, the experiments were repeated using MUC1-positive breast cancer cells, T47Ds, that had been stably transfected with siRNA to knockdown MUC1 expression. T47D cell growth is stimulated by the addition of bivalent Anti-MUC1* and the characteristic bell-shaped growth curve is produced. However, in cells in which MUC1 expression has been suppressed by at least 90%, the antibody has no stimulatory effect (Fig. 3E). Lastly, experiments showed that stimulation of MUC1-positive cells with bivalent Anti-MUC1* induced the phosphorylation of ERK1/2. In contrast, the monovalent Fab fragment of Anti-MUC1* suppressed basal levels of phosphorylated ERK1/2 (Fig. 3F). The phosphorylation of ERK1/2 is a key step in the activation of the MAP kinase signaling cascade. Taken together, these results are consistent with the idea that MUC1* is a growth factor receptor or co-receptor that mediates cell growth via dimerization of the extracellular domain, whereupon the MAP kinase signaling pathway is activated.

### MUC1* activating ligands

**Figure 4:**
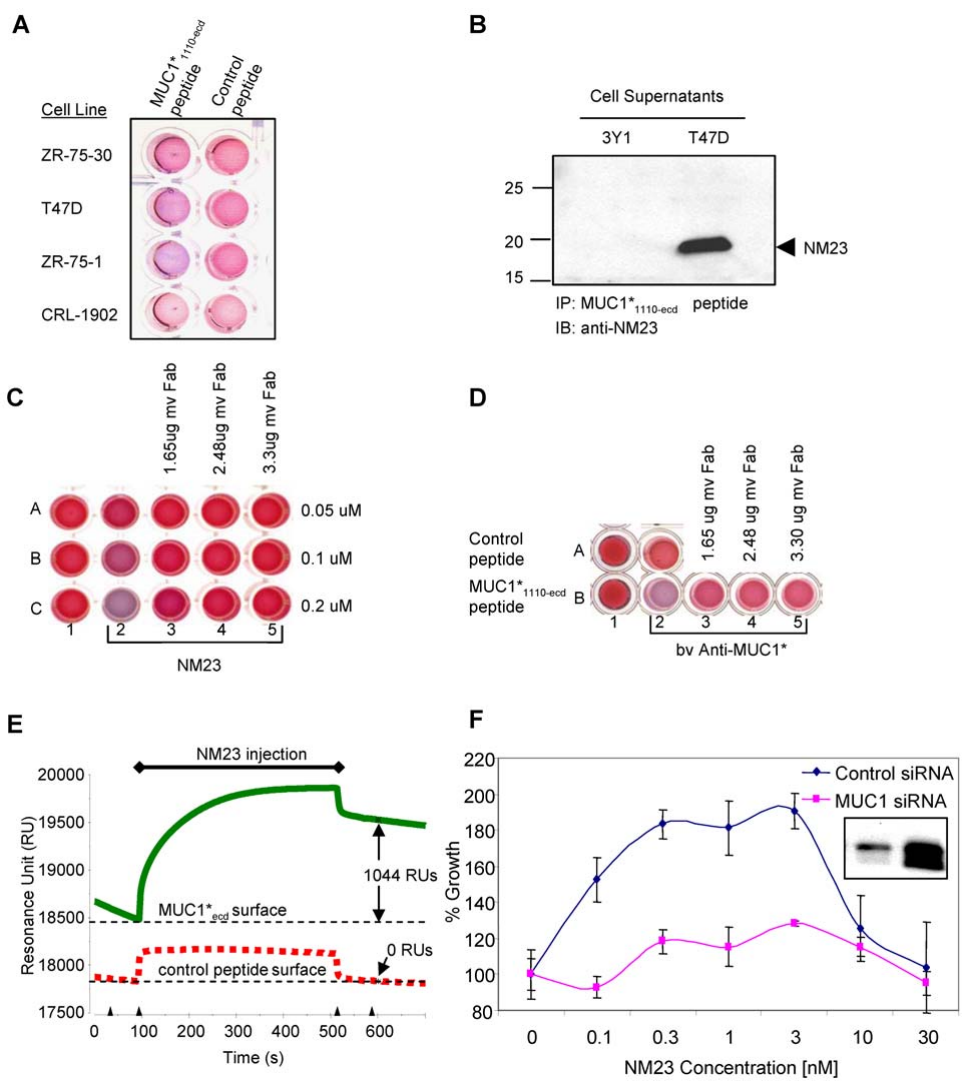
NM23 is a MUC1* ligand that stimulates cell growth. A. Lysate-supernatant mixtures from a panel of MUC1-positive breast cancer cells were tested for the presence of a ligand capable of dimerizing a MUC1*_1110-ecd_ peptide (membrane proximal 45 amino acids of the extracellular domain: 1110–1155). Lysate-supernatant mixtures were incubated with nanoparticles bearing either a control peptide or a MUC1*_1110-ecd_ peptide, histidine-tagged at the C-terminus. A nanoparticle solution color change from pink to a purple/blue indicates that a species in the mixture has simultaneously bound to 2 or more MUC1*_1110-ecd_ peptides. B. Bivalent (bv) Anti-MUC1* (0.77 ug) is added to nanoparticles bearing either a control peptide (Row A) or MUC1*_1110-ecd_, peptides (Row B). Antibody binding to two peptides on separate nanoparticles causes nanoparticle aggregation and the solution color change from pink to purple/blue. In Row A, wells 3–5, this aggregation is inhibited by the addition monovalent Anti-MUC1* Fab (mv Fab). C. NM23 at 0.05, 0.1 or 0.2 uM, (Rows A, B and C respectively) is added to nanoparticles bearing MUC1*_1110-ecd_ peptides (Columns 2–5). Added NM23 induces a color change, presumably as NM23 dimers bind to nanoparticle-immobilized MUC1*_1110-ecd_ peptides. The addition of monovalent Anti-MUC1* (mv Fab), in Columns 3–5, inhibits the color change. D. Conditioned media from MUC1-positive breast cancer cells, T47Ds, and MUC1-negative rat fibroblasts, 3Y1s, was mixed with beads bearing the MUC1*_1110-ecd_ peptide. Immunoprecipitated species were then analyzed by western, wherein the gel was blotted with an anti-NM23 antibody. The western blot shows that T47Ds secrete NM23 while 3Y1s do not. E. Surface plasmon resonance (SPR) was used to detect direct binding between NM23 (15 nM) and MUC1*_1110-ecd_ peptides. The sensogram shows that 1044 RUs of NM23, injected at 15 nM, bound to a surface bearing MUC1*_1110-ecd_ peptides (solid line) but no NM23 bound to a control surface bearing an irrelevant peptide (dashed line). F. Stimulation of T47D breast cancer cell growth by NM23 is dependent upon MUC1 expression. Exogenous NM23 added to T47D breast cancer cells stimulates growth and produces bell-shaped curve indicative of receptor dimerization. Growth is greatly reduced in cells stably expressing MUC1-specific siRNA, compared to cells expressing a control siRNA. Western blot quantifies the siRNA suppression of MUC1.

If MUC1 or MUC1* functions as a growth factor receptor that is activated by dimerization of the extracellular domain, then it follows that MUC1-positive cancer cells may secrete a dimerizing ligand(s). We designed a nanoparticle experiment to screen cancer cell lysates and supernatants for the presence of this ligand. MUC1*_1110-ecd_ peptides (N-1110-1054-C: see Fig. 1B) were attached to gold nanoparticles via a C-terminal histidine tag and lysate/supernatant samples were added [27]. If a dimerizing ligand were present, it would simultaneously bind to a first peptide on a first nanoparticle and a second peptide on a second nanoparticle, thus causing bulk crosslinking of nanoparticles and peptides. Due to an inherent property of gold nanoparticles, crosslinking causes a color change from the characteristic pink to a purple/blue [28]. The addition of the lysate/supernatant mixtures to MUC1*_1110-ecd_ peptide-bearing nanoparticles caused the solution to turn purple/blue. The rate of color change roughly corresponded to the amount of MUC1 that each cell line produced. No color change resulted when the lysate/supernatant mixtures were added to nanoparticles bearing a control peptide (Fig. 4A).

To identify what appeared to be a MUC1* ligand(s), beads bearing MUC1*_1110-ecd_ peptides were used to immunoprecipitate the unknown proteins from the lysate/supernatant mixtures from MUC1-positive cancer cells. The eluates were separated by SDS-PAGE and visualized using silver staining methods. There were essentially only two bands that were precipitated by the MUC1*_1110-ecd_ peptide and not precipitated by a control peptide: a prominent 23 kDa band and a fainter 17 kDa band (Fig. S3). The protein bands were excised from the gel and analyzed using N-terminal microsequencing, which identified them as NM23, isoforms H1 and H2 respectively. NM23 is normally found in the cytoplasm of all cells but is often secreted by cancer cells [29] indicating that it could be a ligand for the extracellular portion of MUC1*. Cell supernatants were immunoprecipitated with MUC1*_1110-ecd_ peptides attached to beads, then analyzed by western blot. The supernatants from MUC1-positive cancer cells, but not the control cells, generated a strong protein band at approximately 23 kDa that reacted with anti-NM23H1 (Fig. 4B). This confirmed that the proteins in the cancer cell supernatants that were immunoprecipitated with MUC1*_1110-ecd_ peptides were indeed NM23.

To detect direct binding between NM23 and MUC1*_1110-ecd_ peptides and to confirm specificity, another nanoparticle experiment was performed. Recombinant human NM23 was added to MUC1*_1110-ecd_-peptide-bearing nanoparticles or nanoparticles bearing a control peptide. The addition of NM23 to nanoparticles bearing MUC1*_1110-ecd_, caused a solution color change from pink to purple/blue and the degree of the color change was a function NM23 concentration. The monovalent Fab of Anti-MUC1* competitively inhibited binding between particle-immobilized MUC1* peptides and NM23 (Fig. 4C). To further demonstrate the specificity of the nanoparticle assay itself, bivalent Anti-MUC1 was added to MUC1*_1110-ecd_-peptide-bearing nanoparticles, which as expected resulted in the pink to blue color change. That binding was also competitively inhibited by the monovalent Fab (Fig. 4D). Together, these results indicate that NM23 directly binds to the MUC1*_1110-ecd_ peptides and that the binding is specific and multimeric.

The binding of NM23 to MUC1* peptides was also demonstrated using a Biacore surface plasmon resonance (SPR) instrument and methods described in Bamdad [30]. SPR techniques measure changes in optical properties at a solution/surface interface when proteins in a flowing solution bind to a target protein that has been immobilized on the surface. Because binding takes place in a constant flow environment, the technique can be used to extract binding constants and affinities. We immobilized MUC1*_1110-ecd_-peptides on the surface and injected NM23 into the flowing buffer. Our experiments showed that purified bovine as well as recombinant human NM23 specifically bound to MUC1*_1110-ecd_ peptide surfaces at concentrations as low as 1 nM, but not to surfaces that presented an irrelevant peptide of similar size (Fig. 4E). Subsequent binding of an antibody that recognizes NM23 verified that the change in optical readout at the solution-surface interface was due to the binding of NM23 to the MUC1*_1110-ecd_ peptide surface. Pico- to nanomolar affinities were measured by analyzing the resultant binding curves with BiaEvaluation software.

We next tested the ability of exogenous NM23 to stimulate the growth of MUC1-positive cancer cells. T47D breast cancer cells were stably transfected with either siRNA that suppressed MUC1 expression or with a control siRNA. If NM23 were an activating ligand of MUC1, we would expect to see more growth stimulation in the cells transfected with the control siRNA than in the cells transfected with siRNA that suppressed MUC1. Exogenous NM23 was added at concentrations in the same range as those shown to bind to MUC1*_1110-ecd_ peptides in the SPR experiments. The addition of exogenous NM23 stimulated the growth of the cancer cells in which MUC1 was not suppressed. A bell-shaped growth curve indicative of receptor dimerization was generated. The siRNA suppressed cells were weakly stimulated. Western blot analysis shows that the siRNA suppressed MUC1 expression by about 75% (Fig. 4F). These results are consistent with the idea that NM23 act as a growth factor for the MUC1* receptor.

### MUC1 protein contains a self-aggregation domain

**Figure 5:**
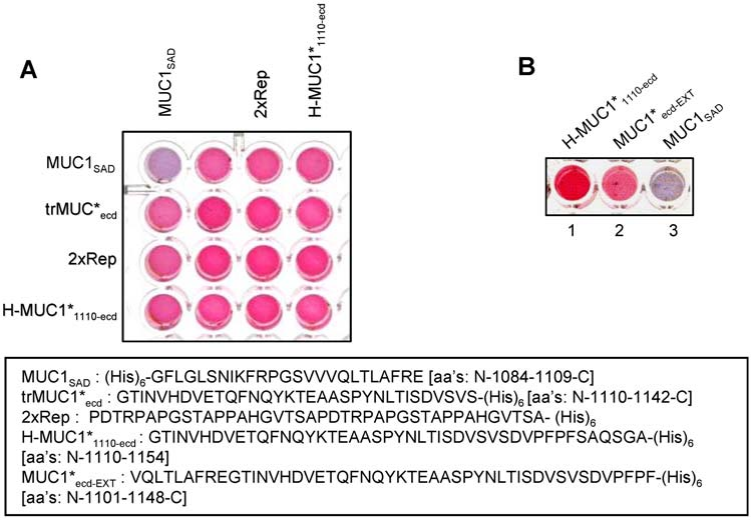
MUC1 has a self-aggregation domain (SAD) immediately N-terminal to MUC1*. A. Degenerate peptides derived from the membrane-proximal extracellular domain of MUC1, as well as a portion of the tandem repeats, were separately attached to pools of nanoparticles. Aliquots of nanoparticles, each bearing a different peptide sequence, were mixed together to test for possible interaction between one peptide and another, or for self-interactions. Interactions among nanoparticle-immobilized peptides are seen as a color change from pink to purple/blue. The well in the upper left corner shows that the peptide MUC1_SAD_ self-aggregates. No other peptides tested bound to each other. B. Wells 1–3 contain nanoparticles bearing histidine-tagged peptides of a single sequence. Well 1, containing MUC1*_1110-ecd_ (N-1110-1154-C) does not self-aggregate as evidenced by the pink solution color. MUC1*_SAD_, (N-1084-1109-C), which is immediately adjacent to MUC1*_1110-ecd_, clearly self-aggregates as the solution color turned a deep purple/blue (Well 3). A third peptide MUC1_ecd-EXT_ (N-1101-1148-C) comprised of 39 amino acids of MUC1*_1110-ecd_ and extending nine additional amino acids into the self-aggregation domain (SAD), albeit to a lesser degree (Well 3).

Our finding that the major species in cultured cancer cells and on cancerous tissues is MUC1*, the cleaved form, led us to wonder if MUC1 cleavage had any functional significance. We designed a nanoparticle experiment to investigate whether or not a portion of MUC1 that is shed from the cell surface after cleavage could bind to the MUC1*_1110-ecd_ peptide as previously suggested [20]. Peptides corresponding to products of proposed cleavage sites were synthesized with histidine-tags and immobilized on gold nanoparticles. Various combinations of possible binding pairs were mixed together to test for interaction. None of the peptides that we tested bound to the MUC1*_1110-ecd_ peptide. However, unexpectedly, we discovered that a portion of the MUC1 protein self-aggregates. A non-repeating 25 amino acid sequence (N-1084-1109-C) that is immediately amino terminal to the MUC1*_1110-ecd_ portion, which we call MUC1_SAD (self-aggregation domain)_, self-aggregates (Fig. 5A). In a follow up experiment, we found that a shifted frame peptide (N-1101-1148-C) consisting of most of the sequence of MUC1*_1110-ecd_ but extending nine (9) additional amino acids into the MUC1_SAD_ region also self-aggregates, but to a lesser degree than the full MUC1_SAD_ (Fig. 5B). Of note, this self-aggregating domain of MUC1 lies within a larger conserved motif called an SEA domain [31].

## Discussion

Our study examined the expression patterns of, and the growth-promoting activities of, full-length MUC1 and its cleavage product, MUC1*. For many years researchers had observed a link between the aberrant expression of MUC1 and cancer. It had also been known for many years that MUC1 undergoes cleavage to generate a low molecular weight fragment, which remains membrane-bound, plus a soluble high molecular weight portion that can be shed from the cell surface. However, studies needed to be done that examined the roles of each fragment individually. To this end, we produced and characterized a novel antibody to the MUC1 protein that is able to distinguish the 20–35 kDa membrane-bound fragment from the remaining 200^+^ kDa amino terminal portion. We call this antibody anti-MUC1*. The use of this novel antibody along with another antibody, VU4H5, that recognizes the high molecular weight soluble fragment, revealed that MUC1* is the predominant species on cultured cancer cells and on tumor tissue and is surface-expressed. Full-length MUC1 is the minor species and its expression appears to be restricted to the cytoplasm. We therefore generated a MUC1* construct, MUC1*_1110_ (having only forty-five (45) amino acids of the extracellular domain) to mimic the species found on tumor tissue. The expression of this minimal MUC1* fragment into MUC1-negative cells is sufficient to stimulate cell proliferation and to render cells resistant to death induced by chemotherapy agents. We further demonstrated that treating cells that express MUC1* with bivalent anti-MUC1* stimulates cellular proliferation and transient phosphorylation of ERK1/2. These results suggest that MUC1* might act as a receptor for an extracellular factor that promotes MUC1* dimerization, activation of the MAP kinase pathway and thus tumor cell proliferation.

Ligand fishing experiments that we performed identified NM23 as a potential ligand for MUC1*. NM23 is normally cytoplasmic but is often secreted by tumor cells [26]. Our in vitro experiments showed that NM23 binds to MUC1* extracellular domain peptides with nanomolar affinity. Further, the addition of exogenous NM23 to MUC1*-expressing tumor cells stimulated growth, while siRNA suppression of MUC1 in those same cells, suppressed that stimulation. Importantly, direct binding between NM23 and MUC1* peptides was detected for the same concentration range at which NM23 stimulated cancer cell growth. NM23 has been simultaneously linked to both metastasis suppression [32] and metastasis promotion [33]. It should also be noted that stimulation of tumor cells with NM23 produces the bell-shaped growth curve that is characteristic of ligand-induced receptor dimerization. Notably, NM23 can be a monomer, dimer, tetramer or hexamer depending on concentration [34] and thus its function may depend on its multimerization state. For example, an NM23 mutant, S120G, which inhibits hexamer formation but preferentially form dimers is common in neuroblastomas [35]. It is possible that mutations or expression levels of NM23 that result in dimers as the major species could drive cell growth by stimulating the MUC1* receptor for prolonged periods of time.

Previous studies reported that MUC1 is a co-receptor for ErbB2, EGFR and FGFR3 [11], [12]. However, our results argue that MUC1* is capable of functioning as an independent growth factor receptor. 3Y1 cells do not express ErbB2 and express very minimal levels of EGFR and FGFR3. Yet, transfection of MUC1*_1110_ into 3Y1 cells conferred growth factor receptor-like activity. The introduction of MUC1*_1110_ stimulated growth, increased survival and drove more cells into the G2/M phase than did the transfection of the empty vector or full-length MUC1.

Our finding that MUC1 has a self-aggregation domain (SAD) will require further study. The self-aggregation domain of MUC1 may promote the clustering of full-length MUC1 and serve to occlude the ligand binding site of MUC1*. Cleavage at a site that releases the self-aggregation domain could render the membrane-attached MUC1* accessible to activating ligands. MUC1's SAD region is a part of a larger, conserved SEA domain [31] that is found on several cell surface proteins and has been shown to undergo self-cleavage. It is unclear as to whether or not self-aggregation is inherently characteristic of SEA domains. Thus, it is tempting to speculate that cleavage and release of a self-aggregation domain may be a conserved mechanism that allows cell surface proteins to change their function.

In summary, we have found that the predominant form of the MUC1 protein on the surface of tumor cells and tissues is a low molecular weight species that we call MUC1*. Treatment of cells expressing MUC1* with a bivalent ligand stimulates tumor cell growth. The construct MUC1*_1110_, truncated after only forty-five (45) amino acids of the extracellular domain, may represent the minimal region of MUC1 necessary for its oncogenic function. Thus, a new class of potent anti-cancer therapeutics could be designed around agents that interfere with the interaction between the extracellular domain of MUC1*_1110_ and its activating ligands. The more immediate implications of our findings are for cancer diagnostics. The aberrant expression of MUC1 has been correlated with recurrence and life expectancy for prostate cancer [36], [37]. These studies were based on measurements of the full-length protein or full-length transcript but were blind to the cleaved form, MUC1*. Our novel antibody, Anti-MUC1*, specifically stains MUC1*, but does not stain the full-length protein on tissues. A biopsy test that quantifies the amount of MUC1* that is accessible to its activating ligands would be a more accurate prognostic indicator than tests that detect portions of MUC1 that are typically shed from the surface of tumor cells.

## Methods

### Cell Lines and Culture

Human embryonic kidney HEK 293 cells, rat 3Y1 fibroblasts and human HCT116 colon carcinoma cells were cultured in DMEM supplemented with 10% heat inactivated fetal bovine serum (HI-FBS), 100 unit/ml penicillin, 100 µg/ml streptomycin, 2 mM L-glutamine and further supplemented with and 600 µg/ml G418 sulfate (HyClone, Logan Utah) to culture transfectants. Breast carcinoma cells T47D, ZR-75-1 (CRL-1500) and ZR-75-30 (CRL-1504) cells were cultured in RPMI 1640 medium containing 10% HI-FBS, 100 units/ml penicillin, 100 µg/ml streptomycin, 2 mM L-glutamine, 1.5 g/L sodium bicarbonate, 4.5 g/L glucose, 10 mM HEPES, and 1.0 mM sodium pyruvate. Medium was further supplemented with 0.5 µg/ml puromycin (CALBIOCHEM) for siRNA experiments.

### Anti-MUC1* Antibodies

Polyclonal: Rabbits were immunized with a MUC1*_1110-ecd_ peptide conjugated at the C-terminus with KLH and affinity purified by column chromatography. Papain digestion, then purification over a protein A column produced monovalent Anti-MUC1*, collected from the flowthrough (QCB). Mouse monoclonal antibodies were generated via immunization with the same peptide.

### Western Analysis

Cell pellets were lysed in cold RIPA buffer plus protease inhibitors. Protein concentration was determined using a BCA Assay Kit (Pierce). 40 µg of lysates were resolved on 6% or 12% acrylamide SDS-PAGE gels. Proteins were transferred to Immobilon P membrane (Millipore) under semi-dry conditions. Membranes were blocked for 1 hour in 5% nonfat dry milk in PBST (PBS containing 0.05% Tween-20) and hybridized overnight in 5% nonfat dry milk in PBST with Anti-MUC1* (1∶500; Minerva Biotechnologies), VU4H5 (1∶200, Santa Cruz), Ab-5 (1∶200, LabVision). Membranes were washed three times in PBST, and secondary detection was done using 1∶10,000 dilutions of HRP-conjugated anti-mouse, anti-rabbit, or anti-Armenian hamster antibody (Jackson ImmunoResearch Labs). Membranes were washed three times, and chemiluminescent detection was done using ImmuneStar reagent (Bio-Rad).

### Deglycosylation

Whole cell lysates from indicated cell lines were incubated for 24 hours in the presence of PNGase, O-glycosidase, and Neuraminidase (New England Biolabs), according to manufacturer's directions.

### Immunohistochemistry of Tissues

Serial sections from formalin fixed, paraffin embedded human tissue specimens were probed with Anti-MUC1* at 0.5 µg/ml or VU4H5 at 1 µg/ml, or irrelevant control antibodies. Specimens were processed using an automated stainer (Dako and LabVision) with Dako products and included antigen retrieval with a low (6.0) pH citrate buffer (Dako). Tissues were obtained from untreated patients.

### Survival Assays

#### Survival of 3Y1 transfected with either MUC1*1110 or full-length MUC1 in the presence of chemotherapeutic drugs

Nomenclature for single cell clones of transfectants is “parent cell name/MUC1 or MUC1*/clone #”. 5 × 10^5^ cells/well were plated into 6 well plates in DMEM with 10% serum and G418. The next day, AraC was added to 3Y1 transfectants to a final concentration of 100 µM. Experiments were performed in triplicate. DMSO was added to control wells. After 30 hours cells were trypsinized and resuspended then treated with trypan blue. Live cells were counted using a hemocytometer.

### Immunoprecipitation

T47D cell lysate (500 µg) was pre-cleared with 25 µl of Protein G Magnetic Beads (New England Biolabs) for 1 hour at 4°C with rotation, and incubated with Ab-5 or Anti-MUC1* antibodies pre-bound to 25 µl Protein G Magnetic Beads for 1 hour at 4°C with rotation. Beads were magnetically immobilized, washed three times with PBS, and eluted by boiling in SDS-PAGE loading buffer for 10 minutes. Precipitated proteins were resolved by 6% SDS-PAGE for Western analysis using VU4H5 antibody and 12% SDS-PAGE for Western analysis using the Anti-MUC1* antibody.

### Immunofluorescence of Cancer cells

3.4 × 10^4^ cells per well were plated in 8-well chamber slides (Nunc) in RPMI with 10% serum (T47D and CRL-1504) or DMEM with 10% serum and G418 (3Y1 and HCT116 transfectants). Next day, cells were washed once in PBS, fixed for 5 minutes with 4% paraformaldehyde in Sodium Cacodylate buffer (0.1 M, pH 7.4). Cells were blocked overnight in 0.25% donkey serum and 0.25% BSA in PBS, and hybridized for one hour in Anti-MUC1* (1∶250 dilution of 0.77 mg/ml solution) and VU4H5 antibody (Santa Cruz; 1∶50 dilution) 0.25% donkey serum and 0.25% BSA in PBS. Cells were washed 3-times for 10 minutes in PBS, and incubated in anti-rabbit Alexa 555 (Invitrogen) and anti-mouse Alexa 488 (Invitrogen) for 30 minutes. Cells were washed 3 times in PBS for 5 minutes. Cover glass was mounted on top of slides with anti-fade mounting medium (Biomeda) and sealed with nail polish.

### Cell Cycle Analysis

HCT116 clones transfected with MUC1, MUC1*_1110_, or empty vector were maintained in DMEM with G418 and 0.5% FBS for 48 hours. Medium was changed to DMEM with G418 and indicated FBS amounts. After 18 hours, cells were washed once in PBS, fixed in 90% Methanol, and stained in PI buffer containing Propidium Iodide, RNase A (Invitrogen), 0.1% Triton X-100 (Sigma) in PBS. Analysis was performed on a FC500 Analyzer (Beckman Coulter) WinCycle32 software (Phoenix Flow Systems), and G2/G1 ratios were calculated.

### MUC1* peptide precipitation and NM23-H1 Western blot

MUC1*_1110-ecd_-His_6_ peptide was added to a final concentration of 2.5 nM in 200 µl of a 5% suspension of Ni-NTA-Magnetic beads (Qiagen) and incubated at 4°C for 1 h. The bead-peptide conjugates were magnetically immobilized and washed 3 times with 500 µl of lysis buffer (50 mM NaH_2_PO_4_, 300 mM NaCl, 10 mM imidazole, pH 8.0). 1 mL of cell culture supernatant from indicated cell lines was added to peptide-bead conjugates, and incubated overnight at 4°C. Precipitate-bound beads were washed 3-times in wash buffer. Precipitated proteins were eluted by boiling in buffer (50 mM NaH_2_PO_4_, 300 mM NaCl, 250 mM imidazole, pH 8.0) for 10 minutes. Samples were mixed with SDS-PAGE sample buffer and boiled for 10 min for resolution on a 4–20% SDS-PAGE gradient gel. Gel was probed with anti-NM23-H1 (SC-343, Santa Cruz).

### Generation of constructs and transfectants

#### 3Y1 and HCT116 MUC1 and MUC1*_1110_ transfectants

Full-length MUC1 cDNA was assembled by piecing together DNA from different EST clones obtained from ATCC (American Type Culture Collection, Manassas VA) and from RTPCR carried out on total RNA from T47D cells. The final coding sequence containing 41 repeats was cloned in between the *EcoR*I and *BamH*I sites of the plasmid pIRES2-EGFP (Clontech). The MUC1*_1110_ expression construct was made by cloning in frame the sequence corresponding to the C-terminal 145 amino acids of MUC1 beginning at GTINV… and ending at …AAAASANL after the nucleotide sequence for the signal peptide. The MUC1 sequence containing constructs and the vector plasmid were transfected into 3Y1 and HCT116 cells and stable transfectants were selected by using G418 at concentration of 600 µg/ml. The pool of transfectants were used to obtain single cell clones by sorting with flow cytometer using VU4H5 (Santa Cruz) for full-length MUC1 stable clones, and MUC1* rabbit polyclonal antibody for MUC1*_1110_ stable clones. Single cell clones were later characterized by FACS and by Western blot.

#### T47D MUC1 siRNA transfectants

T47D cells were stably transfected with recombinant p*Silencer* 3.1-H1 puro plasmid (Ambion, Applied Biosystems USA) containing siRNA inserts using Lipofectin (Invitrogen). The sequence of the MUC1-specific siRNA hairpin was as follows: 5′-GCAGCCTCTCGATATAACC-*ATCTCGAGG*-GGTTATATCGAGAGGCTGC-3′. The hairpin sequence is in italics and the sense and the antisense 19 nucleotide siRNA sequence appears from 5′ to 3′. We used the p*Silencer* puro Negative Control Pasmid supplied by the manufacturer as our negative control for siRNA effect. This plasmid encodes a hairpin siRNA whose sequence is not found in the human, mouse or rat genome databases. Transfected cells were selected with media containing 0.5 µg/ml puromycin. Knock down of MUC1 protein was evaluated by Western analysis using Anti-MUC1* antibody.

### Cell growth assays

#### Clonogenic Assay

Single cell clones of 3Y1 cells transfected with either full-length MUC1, MUC1*_1110_, or empty vector were used. Cells were plated at 1000 cells per 100 mm dish in DMEM media containing 10% fetal bovine serum, penicillin/streptomycin and G418 (600 µg/ml). Cells were grown for 9 days and then fixed in 4% paraformaldehyde for 15 minutes at room temperature. Dishes were washed with water and then stained with 1% crystal violet in 70% methanol for 20 minutes at room temperature. Dishes were washed three times with water and allowed to dry overnight at room temperature. To extract the dye, 10% acetic acid was added to each dish and incubated for 20 minutes at room temperature while shaking. Dye/acetic acid solution was diluted 1∶4 in water and the absorbance was measured at 590 nm.

#### Antibody Stimulation Assays

ZR-75-30 cells, 6000/well, were plated in 96 well plates and zero hour cell count taken the next day when media was changed to 0.1% serum. Anti-MUC1* was added every 24 or 48 hours (noted in figure) and cells were grown until first plate reached 80% confluency. Control antibodies, anti-His (IgG1: Santa Cruz), total mouse IgG (Santa Cruz), monovalent Rabbit anti-mouse IgG Fab (Jackson Immunoresearch) and anti-hemagglutinin (IgG2b: Santa Cruz) were added every 24 hours at the same concentrations as anti-MUC1* antibodies. Control HEK293 cells, 4000/well (faster doubling time) were plated as above. Anti-MUC1* was added every 24 hours until plates were 80% confluent. Monovalent Anti-MUC1* was added to ZR-75-30 cells as described for bivalent antibody with the exception that cells were counted after 144 hours, with antibody added every 24 hours. HEK293 cells were treated with monovalent Anti-MUC1* according to the protocol used by bivalent Anti-MUC1*. All experiments performed in quintuplicate; cells counted on a hemocytometer.

MUC1 siRNA expressing and Control siRNA cells, stably transfected, were plated 2000/well in 96-well plates. After overnight attachment media was changed to 2% serum and zero hour cell count was taken. Anti-MUC1* antibody was added every 48 hours and the final cell count was taken at 72 hours.

#### NM23 growth stimulation assay

T47D cells were stably transfected with either MUC1-specific siRNA or a non-specific siRNA, Control siRNA. Cells were plated 10,000/well, six wells/condition in 96-well plates. After overnight attachment in full serum-containing medium, medium was changed to 3% serum-containing medium and zero hour cell count was taken. Bovine NM23 (Sigma N-2635) was added to a final concentration of 0, 0.1, 0.3, 1, 3, 10, or 30 nM every 48 hours. Cells were counted after five days of NM23 stimulation, and percent growth was calculated relative to the growth of cells to which no NM23 was added. Standard deviations of percent growth are plotted as error bars.

### ERK1/2 activation by MUC1* antibody stimulation

CRL-1500 cells were plated at 7.5 × 10^5^ cells per 60 mm tissue culture dish in 3 ml RPMI. After overnight incubation the cells were serum starved for 48 hours. Then medium was replaced with 2 ml of fresh serum free RPMI, and 2 µg of Anti-MUC1* antibody was added to each plate, except for the negative control. Stimulation was stopped at 5, 10, 20 and 40 minutes by aspirating the media and adding RIPA buffer containing 1 mM Na_3_VO_4_ to the plates. Cells were lysed for 10 minutes and detergent-insoluble material was removed by centrifugation. Equal amounts of protein were resolved on a 12% SDS-polyacrylamide gel and Western analysis was performed using anti-phosoho-p44/42 (Cell Signaling Technology).

### Nanoparticle assays

#### MUC1* - ligand binding interaction

Histidine-tagged MUC1*_1110-ecd_ peptide or irrelevant peptide bearing nanoparticles were prepared by incubating 20 µL of 100 µM solution of the peptide per 100 µL of the NTA-Ni^++^ gold nanoparticle solution. 30 µL of the peptide-bound nanoparticle solution was added into each well of a 96 well plate, along with 40 µL of PBS and 30 µL of lysate/supernatant mixture from MUC1-positive tumor cell lines T47D, ZR-75-1, ZR-75-30 and CRL-1902. Color change was recorded after 3 hours.

#### MUC1*- NM23 interaction

To nanoparticles presenting MUC1*_1110-ecd_ or the irrelevant GDV peptide, 70 µL PBS was added followed by the indicated amount of human recombinant NM23 protein. For inhibition with the monovalent Anti-MUC1* Fab, indicated amounts (Fig. 4C) of the antibody were added immediately before adding the NM23 protein. Color change was recorded after 15 minutes.

#### Recombinant NM23 protein

The protein coding sequence for human NM23 was amplified from NM23 cDNA clones (OpenBiosystems) and cloned into pEXP2-DEST bacterial expression vector using Gateway system (Invitrogen). NM23 plasmid was transformed into BL21 (DE3) pLysS cells and its proteins were prepared using either standard IPTG induction or MagicMedia E. coli protein expression media (Invitrogen). His-tagged proteins were purified using NTA-Agarose (Qiagen) methods using 250 mM imidazole for elution.

#### MUC1* - Anti-MUC1* interaction

Histidine-tagged MUC1*_1110-ecd_ peptides or irrelevant “GDV” peptides (GGDVGDSGGDVGDSGGDVGDSH HHHHH) were attached to NTA-Ni^++^-SAM-coated nanoparticles. Peptide bearing nanoparticles were prepared by incubating 10 µL of 5 µM solution of the peptide per 100 µL of the NTA-Ni^++^ gold nanoparticle solution. 30 µL of the peptide-bound nanoparticle solution was added into each well of a 96 well plate containing 70 µL PBS. Indicated amounts (Fig. 4D) of antibody were next added. For competition experiments, the monovalent Anti-MUC1* Fab was added immediately before adding MUC1* antibody. Color change was recorded after 15 minutes.

#### Self-aggregation

20 µL of each histidine-tagged peptide at 100 µM in PBS, were added to each well of a 96 well plate. 100 µL of gold nanoparticles presenting NTA-Ni^++^ prepared according to the method described in Bamdad [25], [34], were added to wells. Color change of the gold nanoparticles was recorded after15 minutes.

### Surface plasmon resonance

A Biacore3000 instrument and BiaEvaluation software was used. Histidine-tagged MUC1*_1110-ecd_ or irrelevant peptide (HHHHHH-SSSSGSSSSGSSSSGGRGDSGRGDS) were immobilized on separate flow channels of 5.7% NTA-Ni^++^ SAM-coated SPR chips, prepared in our lab as described in Bamdad [27]. 35 µL plugs of NM23, purified bovine or recombinant human, were injected into a constant flow stream of 5 uL/minute and sensograms were recorded. NM23 purified from bovine liver (Sigma N-2635) was diluted in PBS alone. Recombinant human NM23 was produced in our lab and affinity purified over a NTA-Ni^++^ column. The protein was diluted in PBS with 15 mM imidazole, which prevented binding to any exposed NTA-Ni^++^ moieties on the chip surface. The chip surface in each flow channel was used only once. Surfaces were not “regenerated” and for each experiment, NM23 in solution was simultaneously introduced to both the MUC1*_1110-ecd_ peptide immobilized on one flow channel and the control peptide immobilized on a second flow channel. Affinities were measured over a wide range of concentrations using a 1∶1 Langmuir model. Actual affinities may vary as first order kinetics cannot adequately describe this system.

## Supporting Information

Figure S1 Cells transfected with full-length MUC1 are cleaved and yield both MUC1 and MUC1*. Single cell clones of 3Y1 cells transfected with either full-length MUC1, MUC1*1110 or empty vector were analyzed by western blot. Upper gel was blotted with VU4H5 and lower gel was blotted with anti-MUC1*. Nomenclature for clones: “parent cell name/MUC1 or MUC1* or Vect/ clone #”. Lanes 2 and 3 contain cleared lysates of MUC1*1110 clones and show no high molecular weight species. Lanes 4 and 5 are from clones of full-length transfectants and show both the high molecular weight and well as low molecular weight proteins. Lanes 7 and 8 are MUC1-positive breast tumor cell lines for comparison.
(1.86 MB TIF)

Figure S2 Anti-MUC1* bivalent antibodies stimulate growth of MUC1-positive tumor cells control antibodies do not. The growth of MUC1-positive breast cancer cells, ZR-75-30, is stimulated by the addition of bivalent (bv) Anti-MUC1* and inhibited by the addition of the monovalent form, Anti-MUC1* mv. A panel of control antibodies was added: anti-His (IgG1), anti-HA (hemagglutinin IgG2b), Mouse IgG (total IgG) and Rabbit IgG mv, which is the monovalent Fab of a rabbit anti-mouse antibody. The addition of bivalent antibody produces the bell-shaped growth curve that is characteristic of receptor dimerization while the addition of monovalent Anti-MUC1* inhibited growth. The control antibodies whether bivalent or monovalent had no effect.
(1.29 MB DOC)

Figure S3 MUC1* ligand fishing and N-terminal sequencing identifies NM23 as MUC1* ligand. MUC1*1110-ecd or an irrelevant peptide (ARKCSLTGKWTNDLGSNMTHHHHHH) were immobilized on NTA-agarose beads. Cell lysate prepared from roughly 24 × 106 T47D cells prepared in PBS containing (0.1% NP40) was allowed to bind to the each type of peptide immobilized beads, washed extensively and eluted with 250 mM imidazole containing PBS. Aliquots of eluates from the two types of beads, either directly or from TCA precipitates of the eluates, were separated on a 12% SDS-PAGE gel. The protein bands were visualized with silver staining and N-terminal sequence was determined for the protein bands that were specific for the MUC1*1110-ecd immobilized beads.
(8.19 MB TIF)
