# Supplementary figures and images for: A Minimal Fragment of MUC1 Mediates Growth of Cancer Cells
Source: PLoS One. 2008 Apr 30;3(4):e2054. doi: 10.1371/journal.pone.0002054 (PMC2329594; doi:10.1371/journal.pone.0002054)

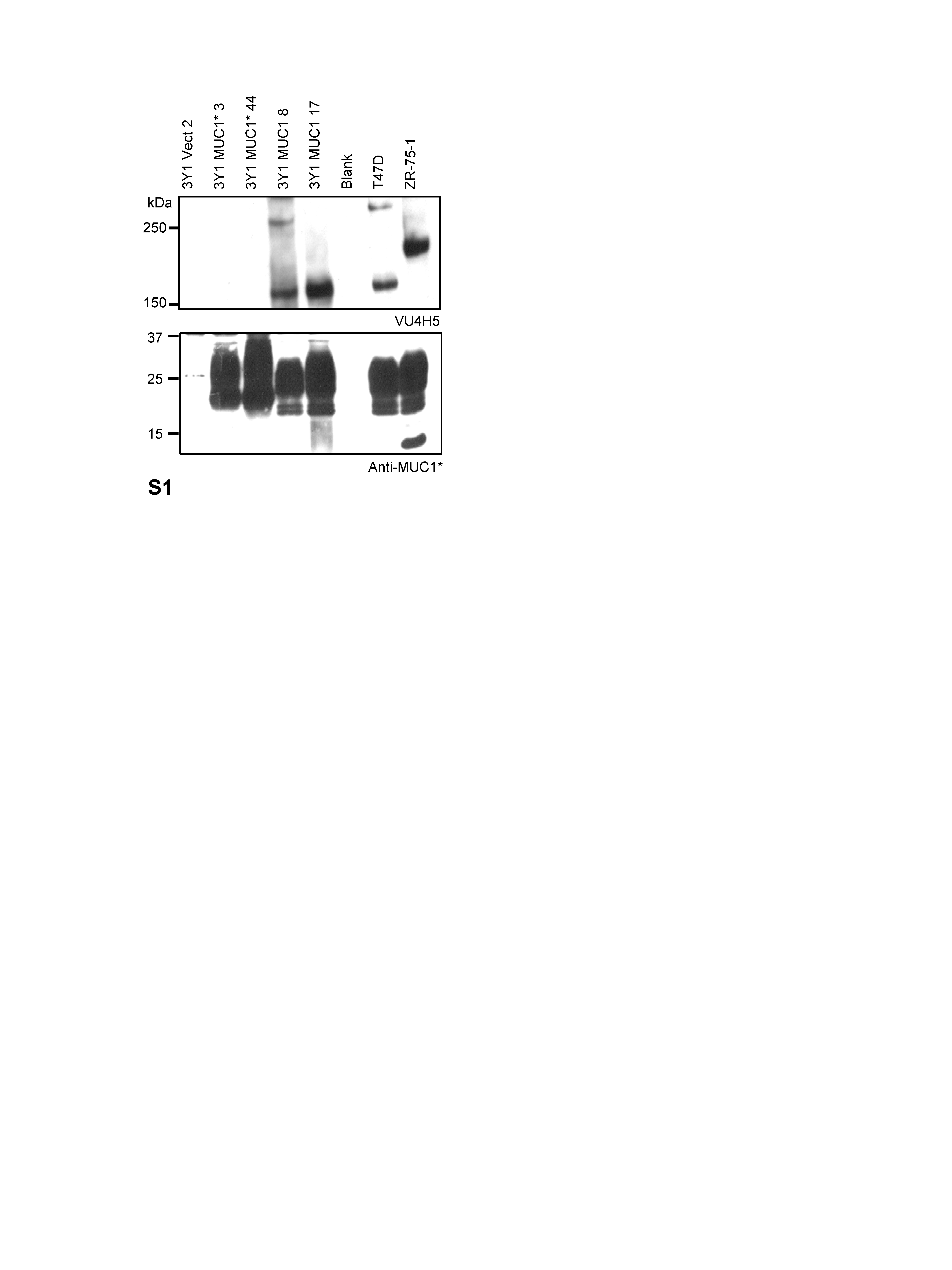

Supplement: Figure S1 — Cells transfected with full-length MUC1 are cleaved and yield both MUC1 and MUC1*. Single cell clones of 3Y1 cells transfected with either full-length MUC1, MUC1*1110 or empty vector were analyzed by western blot. Upper gel was blotted with VU4H5 and lower gel was blotted with anti-MUC1*. Nomenclature for clones: “parent cell name/MUC1 or MUC1* or Vect/ clone #”. Lanes 2 and 3 contain cleared lysates of MUC1*1110 clones and show no high molecular weight species. Lanes 4 and 5 are from clones of full-length transfectants and show both the high molecular weight and well as low molecular weight proteins. Lanes 7 and 8 are MUC1-positive breast tumor cell lines for comparison. (1.86 MB TIF) [file pone.0002054.s001.tif]

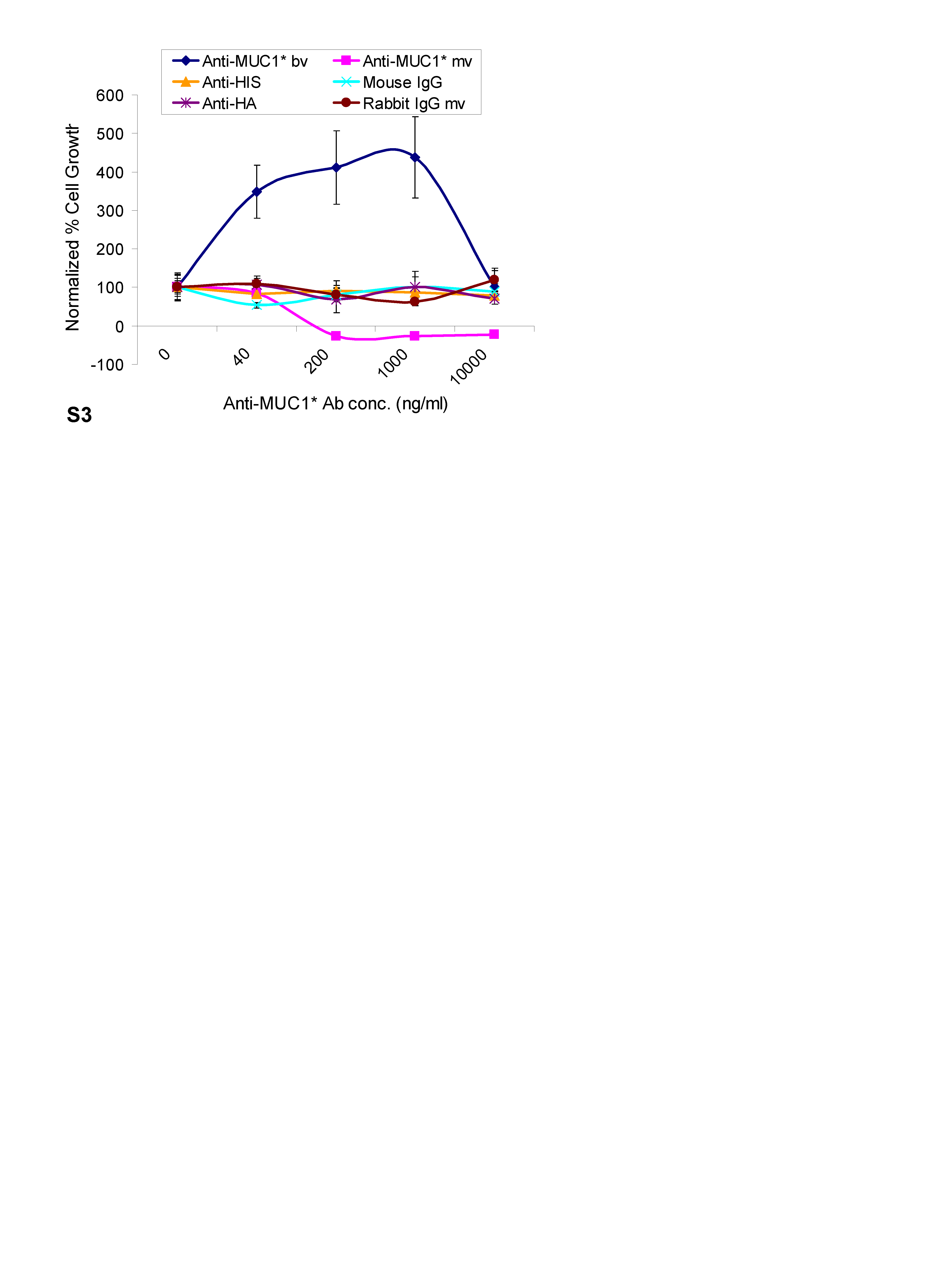

Supplement: Figure S2 — Anti-MUC1* bivalent antibodies stimulate growth of MUC1-positive tumor cells control antibodies do not. The growth of MUC1-positive breast cancer cells, ZR-75-30, is stimulated by the addition of bivalent (bv) Anti-MUC1* and inhibited by the addition of the monovalent form, Anti-MUC1* mv. A panel of control antibodies was added: anti-His (IgG1), anti-HA (hemagglutinin IgG2b), Mouse IgG (total IgG) and Rabbit IgG mv, which is the monovalent Fab of a rabbit anti-mouse antibody. The addition of bivalent antibody produces the bell-shaped growth curve that is characteristic of receptor dimerization while the addition of monovalent Anti-MUC1* inhibited growth. The control antibodies whether bivalent or monovalent had no effect. (1.29 MB DOC) [file pone.0002054.s002.tif]

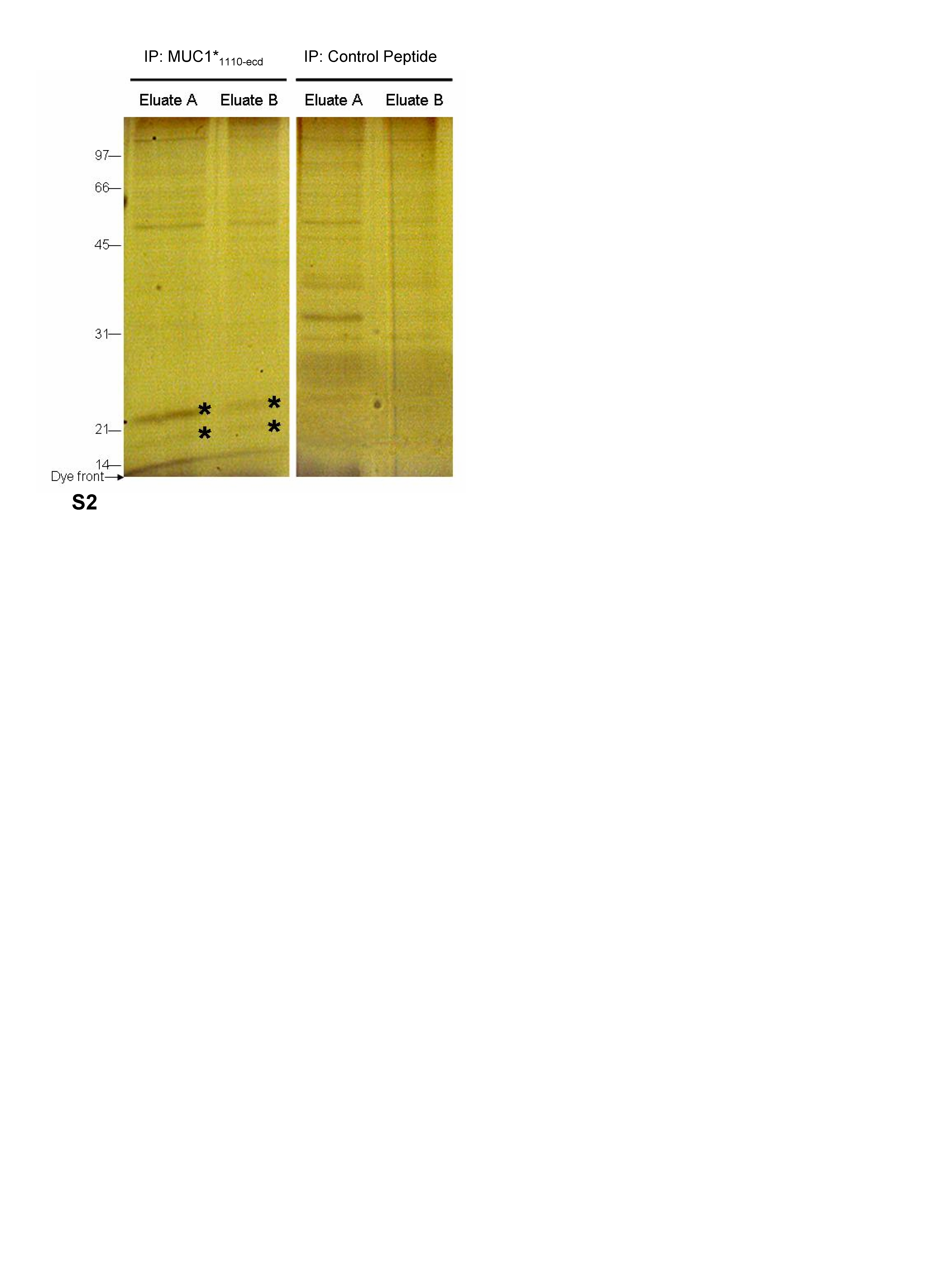

Supplement: Figure S3 — MUC1* ligand fishing and N-terminal sequencing identifies NM23 as MUC1* ligand. MUC1*1110-ecd or an irrelevant peptide (ARKCSLTGKWTNDLGSNMTHHHHHH) were immobilized on NTA-agarose beads. Cell lysate prepared from roughly 24 × 106 T47D cells prepared in PBS containing (0.1% NP40) was allowed to bind to the each type of peptide immobilized beads, washed extensively and eluted with 250 mM imidazole containing PBS. Aliquots of eluates from the two types of beads, either directly or from TCA precipitates of the eluates, were separated on a 12% SDS-PAGE gel. The protein bands were visualized with silver staining and N-terminal sequence was determined for the protein bands that were specific for the MUC1*1110-ecd immobilized beads. (8.19 MB TIF) [file pone.0002054.s003.tif]
